# Development of a Sensor-Based Behavioral Monitoring Solution to Support Dementia Care

**DOI:** 10.2196/12013

**Published:** 2019-05-30

**Authors:** Julia Rosemary Thorpe, Birgitte Hysse Forchhammer, Anja M Maier

**Affiliations:** 1 Engineering Systems Group DTU Management Technical University of Denmark Kongens Lyngby Denmark; 2 Department of Neurology Rigshospitalet, Copenhagen University Hospital Copenhagen Denmark

**Keywords:** ambulatory monitoring, patient-centered care, physical activity, dementia, wearable electronics devices, activity trackers, mHealth, human behavior, system design

## Abstract

**Background:**

Mobile and wearable technology presents exciting opportunities for monitoring behavior using widely available sensor data. This could support clinical research and practice aimed at improving quality of life among the growing number of people with dementia. However, it requires suitable tools for measuring behavior in a natural real-life setting that can be easily implemented by others.

**Objective:**

The objectives of this study were to develop and test a set of algorithms for measuring mobility and activity and to describe a technical setup for collecting the sensor data that these algorithms require using off-the-shelf devices.

**Methods:**

A mobility measurement module was developed to extract travel trajectories and home location from raw GPS (global positioning system) data and to use this information to calculate a set of spatial, temporal, and count-based mobility metrics. Activity measurement comprises activity bout extraction from recognized activity data and daily step counts. Location, activity, and step count data were collected using smartwatches and mobile phones, relying on open-source resources as far as possible for accessing data from device sensors. The behavioral monitoring solution was evaluated among 5 healthy subjects who simultaneously logged their movements for 1 week.

**Results:**

The evaluation showed that the behavioral monitoring solution successfully measures travel trajectories and mobility metrics from location data and extracts multimodal activity bouts during travel between locations. While step count could be used to indicate overall daily activity level, a concern was raised regarding device validity for step count measurement, which was substantially higher from the smartwatches than the mobile phones.

**Conclusions:**

This study contributes to clinical research and practice by providing a comprehensive behavioral monitoring solution for use in a real-life setting that can be replicated for a range of applications where knowledge about individual mobility and activity is relevant.

## Introduction

### Background

The aging of the population and consequent rise in prevalence of conditions such as dementia present a great challenge to society [1]. New care approaches are needed to overcome the increasing disparity between available resources and demands on our health care systems. Mobile and wearable devices, and the rich health-related data these generate, present exciting opportunities to broaden access to care, while enabling predictive, preventive, personalized, and participatory (P4) health care interventions [2]. One interesting avenue is the use of sensor data to monitor behavior. Individual mobility and activity is highly meaningful with regard to independence and quality of life among people with dementia [3] and could be measured using data recorded from smartphones and wearables, such as location, activity, and step count.

### Relevance of Behavioral Monitoring for Dementia Care

Life-space mobility (also referred to as *out-of-home* or *global* mobility) describes the extent to which an individual moves within their environment by any means. Both increasing age and cognitive impairment are associated with reduced mobility [4,5]. This is of great concern with regard to quality of life for people with dementia, as mobility is intrinsically linked to social engagement, functional capacity, affective state and caregiver burden, and a decisive factor for active aging [5-8]. Several factors may be at play when cognitive impairment leads to reduced mobility, such as concerns about safety, usual activities becoming too cognitively demanding, and depressive symptoms (eg, reclusiveness and apathy). Reduced mobility can present a dangerous feedback loop by inhibiting social engagement and stimulation, thereby aggravating the cognitive decline and depressive symptoms that contribute to further mobility reduction. This underscores the importance of maintaining mobility among the elderly and especially the cognitively impaired. Activity can include physical activity levels or activity states or types. Although physical activity is directly linked to mobility, in that a person’s functional capacity contributes to their ability to move in their environment, its measurement also complements out-of-home mobility measures by informing how active a person is while home (or other locations). Monitoring activity is relevant among people with dementia in several ways. For rehabilitation, activity monitoring can guide strategies for increasing engagement in meaningful activities [9] and provide insight into how structured an individual’s daily routines are. Activity levels also provide a useful indicator for functional capacity loss with cognitive impairment. Some studies have even shown a possible association between physical activity and reduced risk of dementia or dementia progression [10].

### Related Work and Open Challenges

Measurement of mobility and physical activity has traditionally been performed using surveys. This approach is limited by its reliance on patients’ memory and subjective perceptions of values such as the distances they cover or time spent active each day, which is especially problematic among people with cognitive impairment. Surveys require input from both patients and health care professionals and thus tend to be restricted to discrete measurements at widely spaced intervals with no information about changes that occur daily or even weekly or monthly. The last decade has seen significant progress toward sensor-based behavior measurement, including among the elderly and cognitively impaired. Mobility and activity features have been calculated using specialized global positioning system (GPS) kits and ankle-worn accelerometers [4,8,11,12]. Although these works offer valuable contributions toward sensor-based behavioral monitoring, the use of specialized systems or strict protocols regarding device placement to measure behavior under experimental conditions is unrealistic for long-term everyday use, therefore difficult to replicate in a real-world setting. This motivates a growing interest in leveraging the wide availability and acceptance of today’s personal devices [8,13-15]. Smartphones and wearables have successfully been applied to measure activity among older adults under free-living conditions [13], daily step count and distance covered among people with dementia [14], and life space among people with Parkinson disease and mild-to-moderate Alzheimer disease [8,15]. System design considerations for real-world use are addressed in a previous study [14], which demonstrates how adequate data are recorded over an extended period (5 months) to reveal behavioral patterns. In some studies [8,13,15], the sensor-based approach is evaluated by comparing measures between experimental and control groups, indicating that significant change in sensor-based behavioral measures might be detected with disease onset/progression; however, no comparison is made with manually reported data. The behavioral measures used vary as follows: activity measures range from daily steps to more detailed descriptions of active and sedentary states; and life space measures range from basic distances to trips or time frames away from home but without extraction of travel trajectories in their estimation. Instead, a threshold distance from home is used to determine whether points are at or away from home. A high threshold (such as 500m in one study [15]) may not detect trips within the subject’s neighborhood. Even with a lower threshold (such as 25m in another study [8]), it is not possible to infer how many places the subject visited if they did not travel home between places, or whether they are continuously moving (eg, going for a long walk) compared with staying at a single location (eg, visiting a friend, in hospital). Without reference data such as self-reports, it is difficult to evaluate the performance of such methods.

This study therefore aimed to advance progress toward mobile/wearable technology–based behavioral monitoring by building upon noted strengths regarding real-world suitability, extending mobility measurement to incorporate GPS trajectory extraction, and providing evidence comparing sensor-derived measures with reference data in the form of self-reports.

### Objectives

The main objective of this study was to develop and test a complete set of tools for measuring mobility and activity using widely available data from off-the-shelf devices. Furthermore, we have described a generic setup for collecting the required data. Together, these are intended to fulfil the purpose of monitoring behavior on an individual level to observe patterns or changes among community-dwelling older adults, such as people with early-stage dementia. Two important goals include

- Transferability: Others should be able to implement the solution at minimal additional effort or expense.
- Real-life suitability: The solution should be developed for long-term, unsupervised, everyday use rather than for laboratory test conditions.

Through fulfilling these objectives, this study contributes a comprehensive behavioral monitoring solution to advance clinical research and practice and enable P4 health care systems.

## Methods

### Extracting Features and Metrics From Behavioral Data

Here we have described the methods used to measure mobility and activity from sensor data, including the algorithms that were adapted and developed for this purpose. We have chosen to focus on 3 types of data: location, step count, and recognized activities, as these are both widely available and highly relevant for measuring mobility and activity. All algorithms and calculations described in this section were implemented in the R programming environment and are available on request*.*

#### Mobility Measurement

Mobility measurement has been described in a number of works on chronic diseases, mental health, and among the elderly [16-19]. These works describe a variety of metrics that we categorize here as spatial, temporal, or frequency-based. Spatial measurements, often termed *life space,* include geographical areas or distances covered, such as the area of the minimum convex polygon (MCP) enveloping a specified quantile of GPS coordinates, distances from home (action range), or total distance covered. Temporal measurements include time spent at or out of home or visiting places of interest. Frequency-based measures include counts within a given period, such as the number of trips out of home or places visited per day or week. Certain life-space measures can be calculated from raw GPS data, whereas other metrics require knowledge of a home location, and some require knowledge of GPS trajectories, that is, the series of stays and moves within the location data.

The raw location data for each user comprises merged watch and phone data, including time stamp, latitude, longitude, and accuracy in meters. The sampling frequency is irregular, and besides any periods of missing data, readings are spaced between a few milliseconds and approximately 5 min apart. The only preprocessing applied was to filter the data according to accuracy with an upper limit of 25 meters. The inputs required to calculate the mobility metrics include the set of coordinate pairs, GPS trajectories (a series of stays and moves), and a known home location. The following sections describe how these inputs are obtained, followed by a description of the metrics calculations.

##### Extraction of Travel Trajectories and Home Centroid

**Figure 1:**
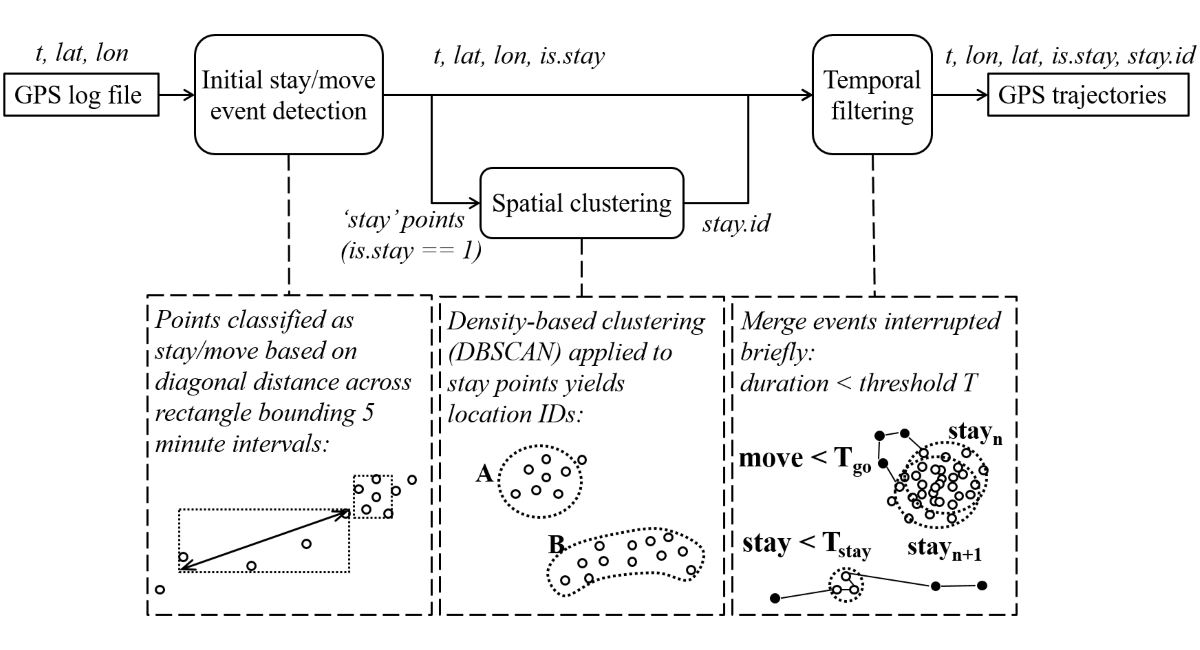
Overview of the trajectory extraction algorithm. DBSCAN: density-based spatial clustering of applications with noise; GPS: global positioning system.

Many temporal and frequency-based measures require GPS trajectories describing how the person stays at or moves between locations, also referred to as *mobility traces* [17]. This requires analysis of the raw location data to extract *stay* (or *stop*, *visit*) and *move* (or *go*) events and identification of geolocations (or *points of interest*, *hotspots*) in the dataset. The identification of trajectories follows a similar approach to those described in the literature [20-22], whereby the data are first split into *stays* and *moves* using time and/or distance thresholds and then clustered to identify geolocations in the dataset. We further included a filtering step to merge temporally close stays or moves that likely belong to the same event. An overview is provided in Figure 1.

**Figure 2:**
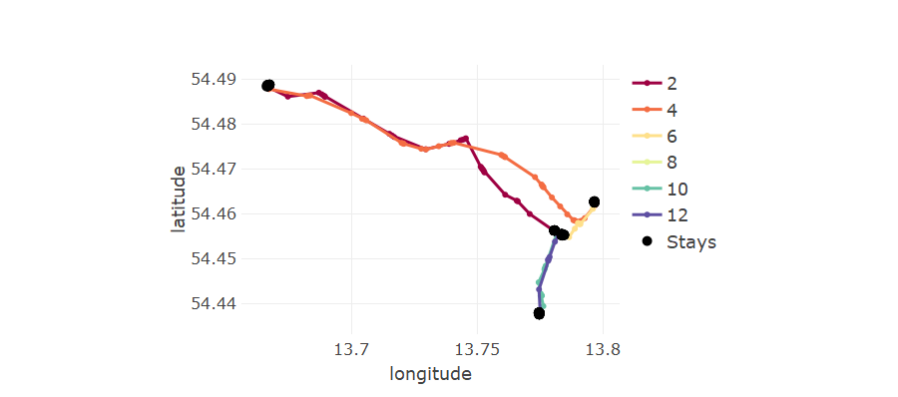
Travel trajectory showing the sequence of stay/move events. All stays are shown as black discs, and each move event shown as a colored line named with its chronological order. The “stays” make up trajectory events 1, 3, 5, 7, 9, 11, and 13. Note: actual GPS data is displaced and the map background removed for anonymity.

The initial stay detection step combines both time and distance information (as in a previous study [22]) to accommodate irregularly sampled location data without the need for further preprocessing. For each location, a rectangle is calculated bounding data from intervals of 5-min ahead. If the diagonal distance across the rectangle exceeds a predefined threshold, the point belongs to a *move*; if below the threshold, all points in the interval are classified as *stay* points. The stay points are then clustered into distinct locations using density-based spatial clustering of applications with noise (DBSCAN), as this method is well-suited to clusters of varying shapes, does not require a priori knowledge about the number of clusters, can handle outliers, and has been successfully applied previously for this purpose [20]. Any outliers not belonging to stay locations are reclassified as move points. In the third (final) step, a time threshold is used to filter very short stays or moves, which is effectively the same as merging move segments or stay events at the same location that are very close together in time.

The algorithm assigns indices to locations without inferring any further information about the nature of the location, with the exception of the subject’s home. The home location is estimated in 2 steps. First, the statistical mode of all GPS points is calculated as *home*. Once all stay locations are extracted from the dataset, those close to home (within a specified threshold) are classified as being at home. An updated home location is then calculated as the centroid of the subset of all points classified as stays at home.

In summary, the GPS log data (time stamp, latitude, and longitude) are used to calculate the following information for each point: whether it is a stay/move; which event it belongs to in a chronologically ordered sequence per day; and for stay points, a location index and whether it is home. An example trajectory is shown in Figure 2.

##### Calculation of Mobility Metrics

The set of mobility metrics was selected by combining various other selections used in the literature for similar purposes [3,8,17,23,24], ensuring that different types of measures are included (ie, spatial, temporal, and frequency-based). The set of mobility metrics includes the following calculations:

- *MCP:* Area of the smallest possible convex polygon constructed around the data, also referred to as the *mobility envelope*. This is calculated by applying the R function, *chull,* to a subset of points for which the distance to the centroid falls within a 99% quantile.
- *Action range:* Straight-line distance between home and the most distal point of a journey, sometimes referred to as *home range*. The geodesic distance is calculated between the home centroid and all other points in the dataset. For each stay and move event in the GPS trajectory, action range is calculated as the maximum of these distances.
- *Distance covered:* Sum of all geodesic distances between consecutive stays centroids.
- *Time spent out:* Sum of durations for all events excluding stays at home.
- *Time spent moving between locations:* Sum of durations for all move events.
- *Number of places visited:* Count of unique places visited (including home). This requires location identifications (IDs) so that a single place is only counted once even when visited multiple times per day.
- *Number of trips:* Count of all moves in the GPS trajectories.

**Figure 3:**
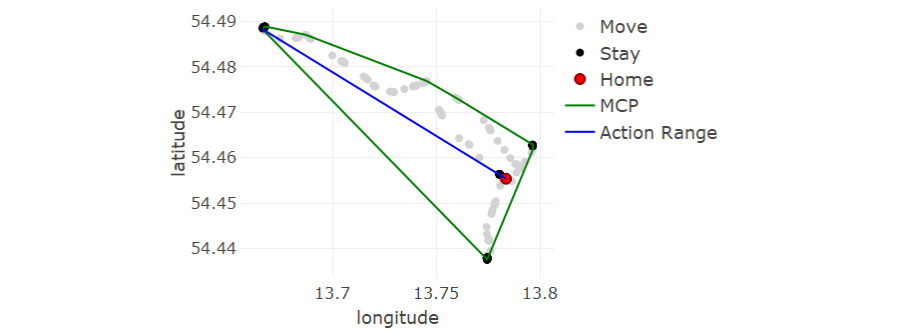
Visual representation of the mobility metrics minimum convex polygon (MCP) and action range, overlaid over global positioning system (GPS) data for one subject for a single day (same trajectory as in Figure 2).

An example of 2 of the spatial measures, MCP and action range, is shown in Figure 3, where these are calculated for the GPS trajectory in Figure 2.

#### Activity Measurement

Within the context of behavioral monitoring for people with dementia, activity measurement is used to gauge how active a person’s everyday life is generally rather than to provide detailed information about physical exercise. Examples of activity measurement in related works (eg, those investigating mobility and activity among similar target groups) include measurements such as active/walking time, number and duration of walking bouts, and total steps per day [7,8,25]. Here we proposed extending the measurement of activity bouts beyond walking (or active) to include other modes of transport, as these offer insight into a person’s everyday routines and preferences, for which any gradual or sudden change could be telling regarding changes in health status. This section describes the methods used to extract these activity features (activity bouts and steps) from sensor data, including recognized activities and step count, respectively.

##### Activity Bout Detection

Activity bouts are detected using data obtained from Google’s activity recognition application programming interface (API: ActivityRecognitionClient). This includes the following types of movement: *still*, *tilting*, *on foot*, *walking*, *running*, *on bicycle, in vehicle*, and *unknown*, where *running* and *walking* are both subsets of *on foot*. Each instance of an activity is recorded with a time stamp and confidence level for its recognition. A number of activities can be recorded at the same time instance. The sampling period is typically approximately 5 min.

**Figure 4:**
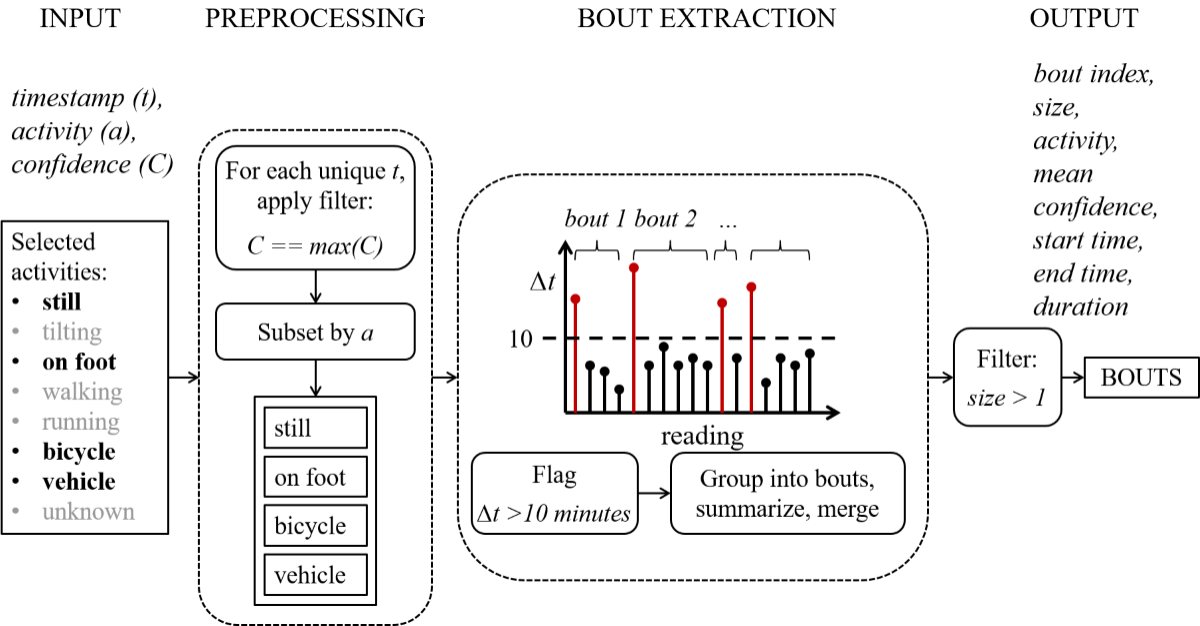
Bout extraction from activity data.

The data are first preprocessed to keep only those activities of interest: *still*, *on foot*, *bicycle*, and *vehicle*. *On foot* is kept in place of both walking and running in accordance with the target group and purpose. The dataset is then reduced further by keeping only those readings with maximum confidence within each distinct time stamp. Where multiple activity types show equal and maximum confidence, all are included. A time threshold is used to split each activity type subset into bouts of continuous activity. Occurrences of the same activity type within 10 min of one another are grouped into the same bout. Bouts comprising only a single reading are filtered out. The bouts are then summarized yielding the following set of measures: bout number (chronologically ordered per day), activity, start time, end time, duration, and number of readings. An overview of the bout extraction algorithm is provided in Figure 4.

##### Step Count

Step count data are used in a straightforward manner to calculate total daily steps. The data are first restructured to obtain a cumulative sum of steps over each day, rather than increasing until the device restarts/reboots. Daily step count from a worn device can potentially account for short ad hoc bouts of activity (such as performing household chores) that take place over and above other exercise regimes or broader movement between geolocations.

### Data Collection Setup: Devices, Sensors, and Apps

We have presented a collection of algorithms and metrics to describe individual mobility and activity. Applying these tools to monitor behavior requires infrastructure to gather the necessary data inputs. In this section, we describe such data collection setup, including devices, sensors, and applications. We have sought to compile a setup that can be replicated by others by using off-the-shelf devices and open-source resources as far as possible.

**Figure 5:**
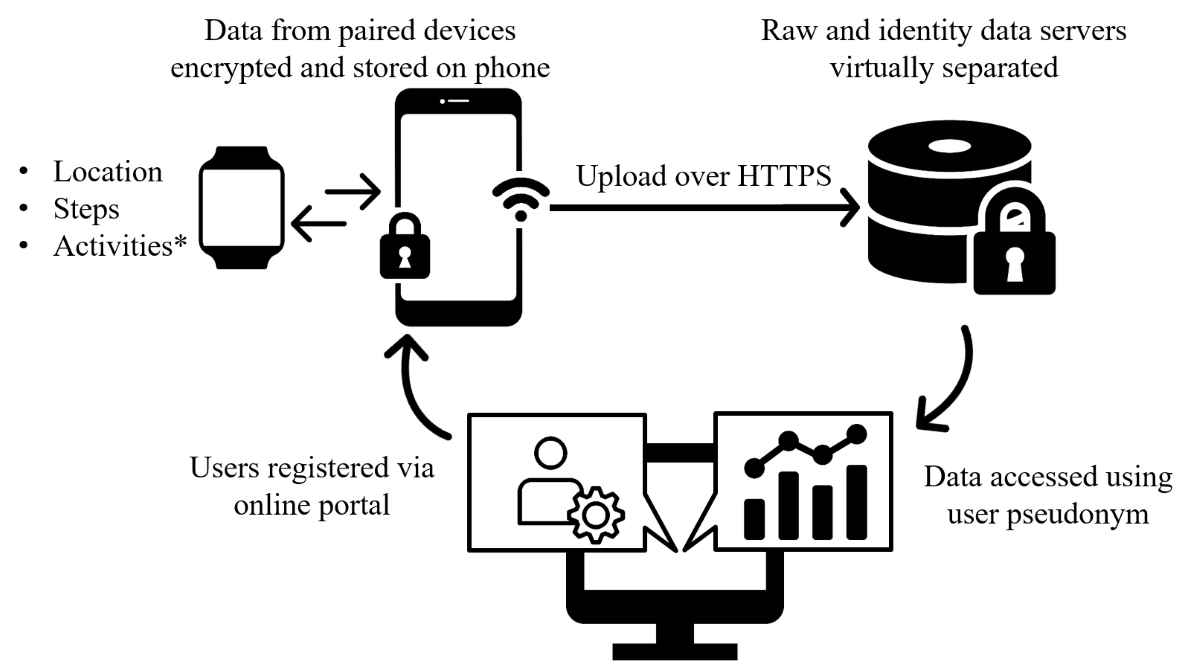
Data collection setup: data is collected using sensors on-board a smartwatch and smartphone (*activities collected using the phone only), encrypted and stored locally on the phone then transferred securely to a server from where it is accessed by an administrator.

The 3 types of data included are location, activity, and step count. Location can be recorded using GPS sensors on board most smartphones and a number of wearables (smartwatches and activity trackers) currently in the market. Recognized activities are calculated primarily from accelerometer data (and in combination with pedometers, gyroscopes, and barometers where available). Step count is typically recorded using activity trackers and smartphones, either from on board pedometers or derived from accelerometer data. The devices used in this study are Google Nexus 5 smartphones and Sony SmartWatch 3 smartwatches, running on Android operating system v6.0.1 and Android Wear operating system, respectively. Android devices offer unrestrictive platforms for development and have been shown to be comparable with ActiGraph for physical activity estimates [26]. Both devices record location and step count, whereas activity types are recorded on the phone only. A custom-built application was used to securely collect, store, and transfer data. The app is an adapted version of that described by Stopczynski et al [27], which is publicly available under the *OpenSensing* GitHub organization. The app uses Google APIs to access sensor data (*LocationListener*, *ActivityRecognitionApi*, and *SensorManager*). New users are registered through a Web portal where they create a front-end username and password and are assigned a back-end pseudonym. The custom app is then accessed from Google Play Store and installed on both paired devices. Watch data are uploaded to the phone, where all data are continuously collected, encrypted, and stored locally on the phone. Only when a Wi-Fi connection is available are encrypted data files uploaded to a server over https. To ensure security, 2 virtually separate servers are employed, an anonymous raw data server and an identity server with user pseudonyms. Data are then decrypted and transferred to a database for analysis as required by an authorized user (eg, a researcher with administrator rights). An overview of the data collection setup is provided in Figure 5.

Through combining the data analysis methods presented in the previous section together with the data collection setup presented here, we provide the necessary tools for a complete behavioral monitoring solution to be applied directly in clinical research and practice. This is evaluated, and the results presented in the following sections.

### Evaluation

The behavioral monitoring solution was implemented in a pilot feasibility study with 5 healthy volunteers (3 female and 2 male), aged between 31 and 40 years. The purpose of the evaluation study was to test the setup under free-living conditions to obtain real-world behavioral data with which to examine the performance of the feature extraction algorithms. Although the intended target group is ultimately people with dementia, the solution is tested among adults with no cognitive impairment to ensure reliable self-reporting, before carrying out any further testing among the target group in future.

#### Equipment, Material, and Methods

Participants were provided with the behavioral monitoring setup, including a smartphone and smartwatch, to use for a period of 1 week. They were instructed to try to wear the watch during the day and charge it at night as required. All participants continued to use their own smartphone during the study and did not interact with the study phone besides taking it with them when going from place to place in everyday life and keeping it charged and paired with the smartwatch. Participants were also provided with a set of log sheets in which they had to record their movements daily. The log sheets comprised 15-min intervals with columns for stay, move, and the stay location or mode of travel.

#### Data Analysis

##### Mobility Measurement

Time charts were created to compare participants’ logs with algorithm results. Results of the trajectory extraction algorithm were mapped to daily time charts as a signal indicating when the participant was in a *stay* or *move* state. Participants’ log sheets were then captured and processed to create matching time charts for their travel trajectories. These were assessed to determine the level of agreement between the logged and reported travel, in terms of both whether trajectory events are detected or not and their timing. The mobility metrics *time outside of home* and *number of places visited* were calculated using both log sheets and the algorithm results and compared. These metrics were selected as they cover different types of information (time and count) that requires both durations and location identification and could feasibly be calculated from participant-reported data.

##### Activity Measurement

**Figure 6:**
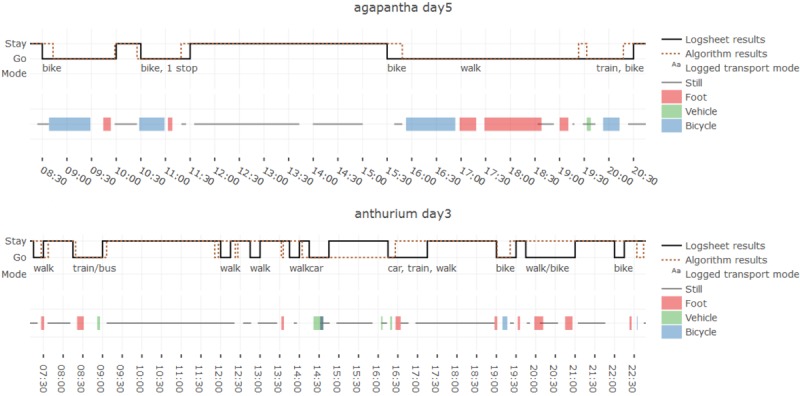
Example time charts for one day from two participants comparing log sheets and algorithm results for trajectory extraction and activity bout detection.

Automatically extracted activity bouts were plotted on the daily time charts (alongside stay/move signals). Each *move* epoch from the trajectories was annotated with participants’ reported transport modes for comparison (see Figure 6). These combined time charts were assessed to gauge the level of agreement between sensor-derived and logged transport modes, identify recurrent errors, and infer strengths or weakness of the approach. Step count data were collected from both the smartwatch and smartphone to compare the 2 sources in terms of daily totals and cumulative step count signals.

## Results

### Mobility Measurement

Participants’ log sheets were compared with results obtained automatically from sensor data. The trajectory extraction algorithm achieved 92% sensitivity for detecting a *move* event. As participants reported that log sheet times were often only approximate estimates (the log sheets could not always be filled at the time of the move), a window of 30 min surrounding the logged *move* was used to determine the correct detection (true positive) by the algorithm.

**Figure 7:**
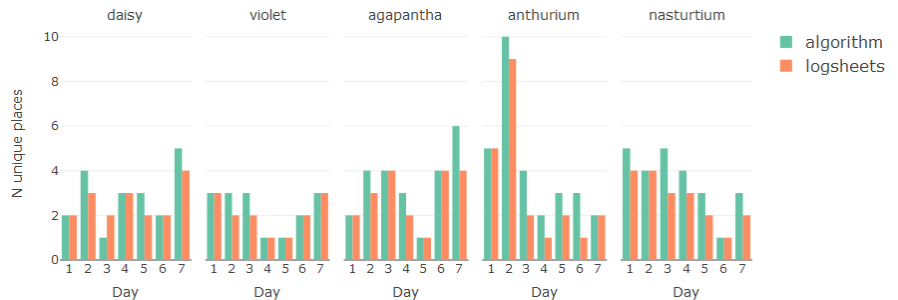
Comparison between the numbers of unique places detected from sensor data and reported by study participants in logsheets.

**Figure 8:**
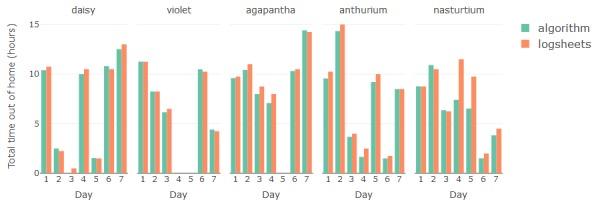
Comparison between the time spent out of home detected from sensor data and reported by study participants in logsheets.

The results for estimation of mobility metrics *number of unique places visited* and *total time spent out* are compared in Figure 7 and Figure 8. Residuals between the algorithm- and log sheet–based estimates offer insight into the performance of the algorithm in relation to manual reporting. The root mean square deviation (RMSD) indicates that the overall difference between algorithm and log sheets is <1 place visited and <1 hour away from home per day (Table 1). The residual means indicate that the algorithm tends slightly toward more places than logged and tends slightly toward less time at home.

Example time charts comparing reported and extracted travel trajectories are shown in Figure 6. These provide further insight into where and how the algorithm deviates from the log sheets. Time offsets between the 2 signals for move start/end times and durations tend to be below 15 min, which is the time resolution of the log sheet data. Certain moves are interrupted by a short stay in either one of the signals and not the other, which could be attributed to, for example, waiting for or changing between transport modes, or stopping to look in a shop. In very few cases, the disagreement between algorithm results and logged data corresponds to longer periods or certain events are missed entirely (eg, in Figure 6, *anthurium day3*, where a 1.5-hour stay is missed). This could be because of signal loss when the participant goes indoors. Conversely, certain moves are detected that are not logged, which may be because of the participant forgetting to report a move or moving very near to their stay location (eg, in their garden).

**Table 1:** Comparison between metrics calculated from log sheet and algorithm trajectories. Residuals are calculated by subtraction of log sheet results from algorithm results, and are used to calculate root mean square deviation (RMSD) and mean (SD).

Residuals (algorithm-logs)	RMSD	Mean (SD)
Number of places per day	0.94	0.60 (0.74)
Time out of home per day (min)	58.66	−25.87 (53.42)

### Activity Measurement

#### Activity Bout Detection

**Figure 9:**
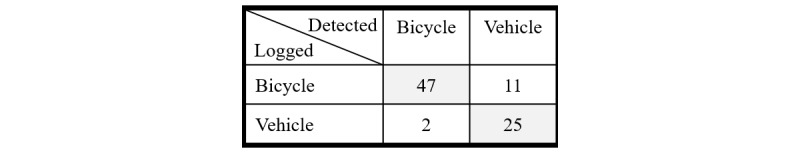
Classification matrix showing confusion between detection of bicycle and vehicle activities in number of occurrences.

The detected activity bouts were plotted together with trajectories in the time charts and compared with reported transport modes. Time charts were initially reviewed to assess overall performance and pinpoint recurring errors to investigate in further detail. The time charts showed that the algorithm generally detects activity bouts during moves and is able to detect multimodal travel, with only limited examples where no bouts are detected during reported travel. One evident limitation is that the detected activity bouts tend not to fill the entire duration of the travel. Instead, each journey comprises one or more shorter bouts along with still periods and gaps in the signal. A recurring error was confusion between travel by bicycle and by vehicle, for which the classification accuracy is determined by Google’s activity recognition. This was investigated further, particularly as the difference between travel by bicycle and vehicle has implications for measuring physical activity or illness-related changes in transport preferences (eg, compared with confusion between bicycle and on foot). Figure 9 shows a classification matrix subset to include only classification between bicycle and vehicle, which indicates accuracy of 85% for classification between the 2 transport modes.

A further observation was a lack of walking bouts during stays, which might be expected as part of an ordinary workday. One contributor may be the filtering of single-reading bouts, presenting an apparent trade-off between detecting more actual activity bouts and introducing additional, incorrect activities during others. Another likely cause is that the data were collected from the smartphone, which may not have been carried on the participant’s person for shorter walking trips around the home or workplace, highlighting the importance of investigation within real-life settings. This is examined more closely in the step count data, which is available for both the phone and watch.

#### Step Count

**Figure 10:**
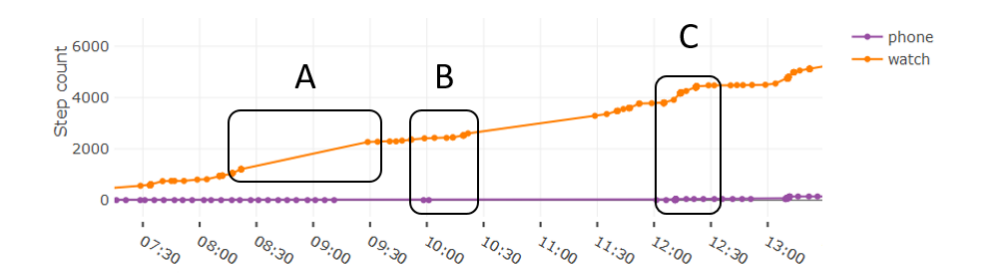
Comparison of step count accumulated over the day between watch and phone. Outlined sections show A) missing updates in watch step count, B) watch records steps while phone does not, and C) watch step count increases at a faster rate than phone.

**Figure 11:**
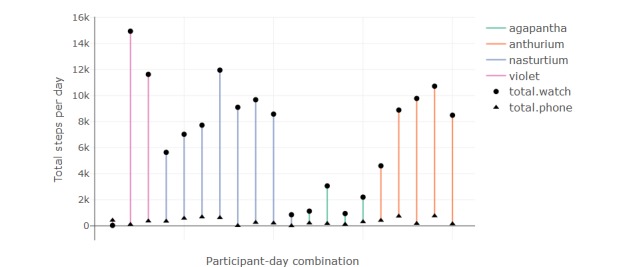
Error bars demonstrating the large difference between total daily step count between watch and phone for each available day.

Step count is compared between the watch and phone for all days where both sources are available. Owing to technical failures in accessing the watch data, it is available for selected participant-days only (agapantha=4, anthurium=5, daisy=0, nasturtium=7, and violet=3). Step count measured from the watches was substantially higher than from the phones. The cause of the disparity between counts appears to be twofold. There are periods where only the watch step count increases and not the phone (Figure 10, section B), indicating that the watch may be worn while the phone is placed still. There are periods where both increase simultaneously, where the watch steps increase at a faster rate than the phone (Figure 10, section C). The substantial difference between daily total steps measured from the watch and phone is demonstrated in Figure 11.

An important limitation evident in the step count data is regarding sampling irregularities. Long periods (over an hour) without any readings are common in the watch step count (Figure 10, section A). Although the steps appear to be logged during these periods (and therefore not expected to influence daily total step counts), this does not support bout detection from the step count data, as it is impossible to infer the distribution of walking bouts over the course of the day.

## Discussion

### Principal Findings

This study has presented a behavioral monitoring solution that leverages widely available data (location, activity, and step count) from mobile and wearable devices used in everyday life. A set of tools was developed to measure mobility and activity, and a generic data collection setup was described to support its implementation. Evaluation of the behavioral monitoring solution showed that it is capable of estimating participants’ travel trajectories from raw GPS data to calculate spatial, temporal, and count-based mobility metrics. An activity bout detection algorithm was shown to successfully extract bouts of activity during travel, including for multimodal transport; however, it appears not to capture their full duration. Step count was shown to be more reliable from a wearable device than from a smartphone under real-world conditions, in which case it is preferable over activity bouts for estimating general daily activity, including during stays such as at work or home.

These results build upon research toward sensor-based monitoring of mobility and activity among older adult populations in [8,13-15] by incorporating trajectory extraction into the mobility measurement, as well as information about modes of transport in activity monitoring, and by providing evidence comparing the sensor-based approach with self-reports. An important goal has been to describe a solution that can be replicated by others for monitoring behavior to support clinical research and practice in a range of applications. The following sections discuss the extent to which the setup is transferable in terms of technical and practical considerations, and of clinical utility.

### Technical Feasibility and Real-World Considerations

A core contribution of this study is the set of algorithms developed to translate sensor data into meaningful behavioral insights. For these to be replicable and useful, we first consider what data are required as inputs and how this is acquired.

The mobility monitoring uses GPS data, which can be acquired using a wide range of available mobile phones. The sampling frequency need not be regular and depends on anticipated trajectory event duration, with a trade-off regarding power consumption. In this study, the mode of sampling periods for location data is approximately 5 min, as it is not of interest to capture stay/move events of shorter duration. Participants were instructed to charge the device overnight, which was sufficient in this study (although it is noted that the devices were not used for other purposes than for data capture). Requirements for location accuracy depend on the anticipated geographical span of stay locations or moves of interest, which in this study was restricted to 25 meters. The activity bout detection relies on Google’s activity recognition (though this could also be implemented using output from another activity recognition algorithm). Step count data are used only for daily totals, which can be obtained from a range of available devices; however, the results in this study indicate that devices vary substantially in step count estimations.

This study has used devices running Android and Google APIs to access the sensor data these generate. This can be replicated using any Android device with the same sensors. The infrastructure for storing and accessing data is based on open-source software from the OpenSensing GitHub organization described in [27] (for Apple products, a similar approach could be feasibly implemented using frameworks such as Apple Research Kit).

This study has endeavored to capture real-world data from everyday life, which leads to important considerations regarding data availability and quality. Data availability from the phone was high, with readings recorded approximately every 5 min throughout the recording period. However, data availability from the watch was poor, with extensive periods (up to days) of missing data. This could be partly attributed to a lost connection between the phone and watch, as the collection of watch data relies on an additional step (compared with phone sensors), whereby data are transferred over Bluetooth to the phone. With regard to data quality, it is not possible to control device placement or wear time, which can lead to missed steps, activities, or trajectory events. This is particularly relevant for counting steps during stay events, for example, to estimate short bouts of physical activity during work, for which a wearable device is recommended.

### Clinical Utility

The transferability and modular structure of the behavioral monitoring approach offers broad potential for supporting clinical research and practice, for example, to evaluate the effect of an intervention on behavior, to monitor behavioral change as an indicator of disease progression, or to advance understanding of how different factors or conditions are associated with changes in behavior.

To better gauge clinical utility, algorithm performance is discussed in relation to intended purpose. The first algorithm uses location data to derive metrics for monitoring life space mobility. Existing methods for assessing life space mobility include a questionnaire-based approach that asks about travel within a set of ranges from home, the frequency (in days per week) at which the subject moves in each range, and about independence level (ie, support from equipment or people) [28]. The algorithm is not able to detect the lowest 2 mobility levels included in the questionnaire: movement within the room in which the subject sleeps and movement to other rooms in their place of residence. It is also not possible to infer the level of support using the algorithm-based approach. However, compared with the questionnaire, the algorithm-based approach offers other types of mobility measures besides distance from home (such as time spent at home or unique locations visited), does not rely on the subject’s ability to recall events, and is more scalable as it does not rely on manual reporting.

Algorithm performance holds implications for the detectable increments of change in behavior. Although there is limited evidence available quantifying change in life space mobility with disease progression among people with dementia, significant differences in mobility between older adults with and without mild-to-moderate Alzheimer disease are documented in [8] using a similar approach. These include differences between group means for an area of approximately 50 km^2^, perimeter of approximately 15 km, distance from home of approximately 1 km, and time away from home of approximately 50 min (6.6% with a reported mean recording time of 7.5 hours per day), where the first 3 are significant and the latter marginally significant. For the spatial measures, such increments should be detectable given the availability, accuracy and sampling frequency of the location data, and trajectory detection presented. For time away from home, the algorithm results deviated from subject reports by >50 min; however, as reporting errors may be a contributing factor, further investigation is necessary to determine whether the algorithm is adequately sensitive to detect changes under 50 min.

The sensor-based activity monitoring approach can be used to gain insight into how a person travels around and to estimate total daily steps as an indicator of overall activity level. Owing to the large discrepancy between device step counts in this study, it is recommended that a device with validated step count is used, or at least that the same device is used throughout any monitoring period. An existing instrument for assessing physical activity is the Global Physical Activity Questionnaire (GPAQ) [29]. Compared with the GPAQ, the sensor-based approach described in this study does not distinguish between activity intensities nor precisely measures time spent in activity states but offers daily step count as an alternative measure of overall daily activity. The GPAQ asks about modes of transport in days per week and time for those days to infer activity performed while moving from place to place. The sensor-based approach is similarly able to measure transport habits; however, as the duration is not precisely measured, this may be better suited to monitoring everyday life habits to detect a change in lifestyle (eg, increase in vehicle use and decrease in walking), rather than, say, adherence to an exercise program.

On the basis of these characteristics with regard to both mobility and activity, suitable target groups could include older adults who live in the community at a baseline functional level sufficient for independent journeys beyond the home. The algorithm-based approach is particularly beneficial where self-reporting is challenging or inaccurate, such as for cognitively impaired individuals. Other relevant application areas in which mobility- and activity-related behavior is highly meaningful include depression or other mental illnesses, active aging, rehabilitation, and lifestyle-diseases (or risk thereof).

### Implications for Advancing Predictive, Preventive, Personalized, and Participatory Health Care

By relying on common, personal devices and allowing for real-world data, the behavioral monitoring solution is geared to enable P4 health care approaches by generating information within the scale and context that this requires. Continuous behavioral monitoring in a home setting can reveal patterns in behavior and fuel the development of predictive models to anticipate disease trajectories or adverse events. This also allows health care professionals to proactively prevent problems earlier than would be possible with prescheduled visits months apart. Knowledge about behavior and lifestyle can inform personalized interventions that take into account which aspects of quality of life are most important to the individual. Data describing behavioral patterns also offer a valuable resource for sharing information about patient status between patients and health care providers in participatory care approaches.

### Conclusions

This study has described a novel sensor-based approach to behavioral monitoring for use among people with dementia. We have presented a set of algorithms to measure mobility and activity from sensor data, including location, recognized activity, and step count data and a technical setup for collecting these data inputs.

An evaluation of the behavioral monitoring solution among 5 participants for 1 week showed that the setup was capable of extracting travel trajectories, mobility features, activity bouts, and daily step count using a smartphone and smartwatch in a natural setting. Each set of results provides related yet distinct information about a person’s daily life: mobility describes the extent to which the persons goes out, activity bouts describe *how* they go out, and step count supplements this with information about how active they are generally, including periods at home or work. Combining these measures provides insights into daily rhythms or longer temporal patterns. This could support clinical applications involving patient groups for whom mobility and activity behavior is closely tied to intervention outcomes, such as among the elderly and people with dementia, and to advance P4 health care.

## Abbreviations

API: application programming interface
DBSCAN: density-based spatial clustering of applications with noise
GPAQ: Global Physical Activity Questionnaire
GPS: global positioning system
MCP: minimum convex polygon
P4: predictive, preventive, personalized, and participatory
RMSD: root mean square deviation
